# Overwhelming COVID-19 Sepsis in a Patient With Idiopathic Pulmonary Fibrosis

**DOI:** 10.7759/cureus.9320

**Published:** 2020-07-21

**Authors:** Ayesha Akram

**Affiliations:** 1 Internal Medicine, Rawalpindi Medical University, Rawalpindi, PAK

**Keywords:** idiopathic pulmonary fibrosis, covid-19, severe sepsis

## Abstract

The new disease caused by severe acute respiratory syndrome coronavirus 2 (SARS-CoV-2) was dubbed coronavirus disease 2019 (COVID-19) by the World Health Organization and declared a pandemic. Initially thought to be a pathogen that primarily attacks the lungs, SARS-CoV-2 has turned out to be a much more formidable foe impacting almost every organ and system aggressively. I report the case of a 60-year-old man who came to the ED due to symptoms of high fever, headache, mild confusion, dry cough and exacerbated dyspnea since the last 24 hours. He had a history of idiopathic pulmonary fibrosis (IPF) and was undergoing treatment with antifibrotic medication. Apart from IPF, he had no other comorbid. He acquired SARS-CoV-2 infection by close contact and infection deteriorated into pneumonia and septic shock. Complete blood count showed white blood cells at 3.3×10^3^/μL and platelets at 71×10^3^/μL; lymphocyte count was low. Arterial blood gases revealed metabolic acidosis. Definitive diagnosis was by a positive real-time reverse-transcriptase-polymerase-chain-reaction (RT-PCR) assay of nasal and pharyngeal swab specimens, and high-resolution computed tomography (HRCT) finding of new-onset ground-glass opacities on the very first day of admission that was the presenting day. The patient became unresponsive and died overnight. As numbers of COVID-19 show an uprise, this case highlights that IPF patients with relatively advanced age need to exercise extra caution because they are at high risk for developing overwhelming COVID-19-linked sepsis, which may be fatal.

## Introduction

Idiopathic pulmonary fibrosis (IPF) is a chronic fibrosing interstitial lung disease (ILD). The precise etiology of IPF is unclear although risk factors include old age and cigarette smoking, the onset is insidious and the prognosis is poor [1,2].

A new disease broke out in Wuhan, China in December 2019 as a cluster of pneumonia cases with etiology unknown [3]. As more unfolded, it was dubbed coronavirus disease 2019 (COVID-19) by the World Health Organization on 11 February 2020, and the causative 2019-novel coronavirus named severe acute respiratory syndrome coronavirus 2 (SARS-CoV-2) [4]. The implications extend outside the lungs; SARS-CoV-2 can lead to septic shock, acute kidney injury, and disseminated intravascular coagulation (DIC) in some of the most severe cases. Signature laboratory findings in severe COVID-19 include lymphocytopenia, thrombocytopenia, leukopenia, elevated C-reactive protein, alanine aminotransferase, aspartate aminotransferase and D-dimer. Old age (IQR 40 to 65 years) and underlying diseases including chronic disease of lung predispose to severe COVID-19 [5].

Here I describe a case of an old man with preexisting IPF and laboratory-confirmed SARS-CoV-2 by real-time reverse-transcriptase-polymerase-chain-reaction (RT-PCR), who presented acutely with rapid progression (within hours) to septic shock.

## Case presentation

A 60-year-old man was brought to the ED with a one-day history of high fever, headache, mild confusion, dry cough, and increasing shortness of breath.

**Figure 1:**
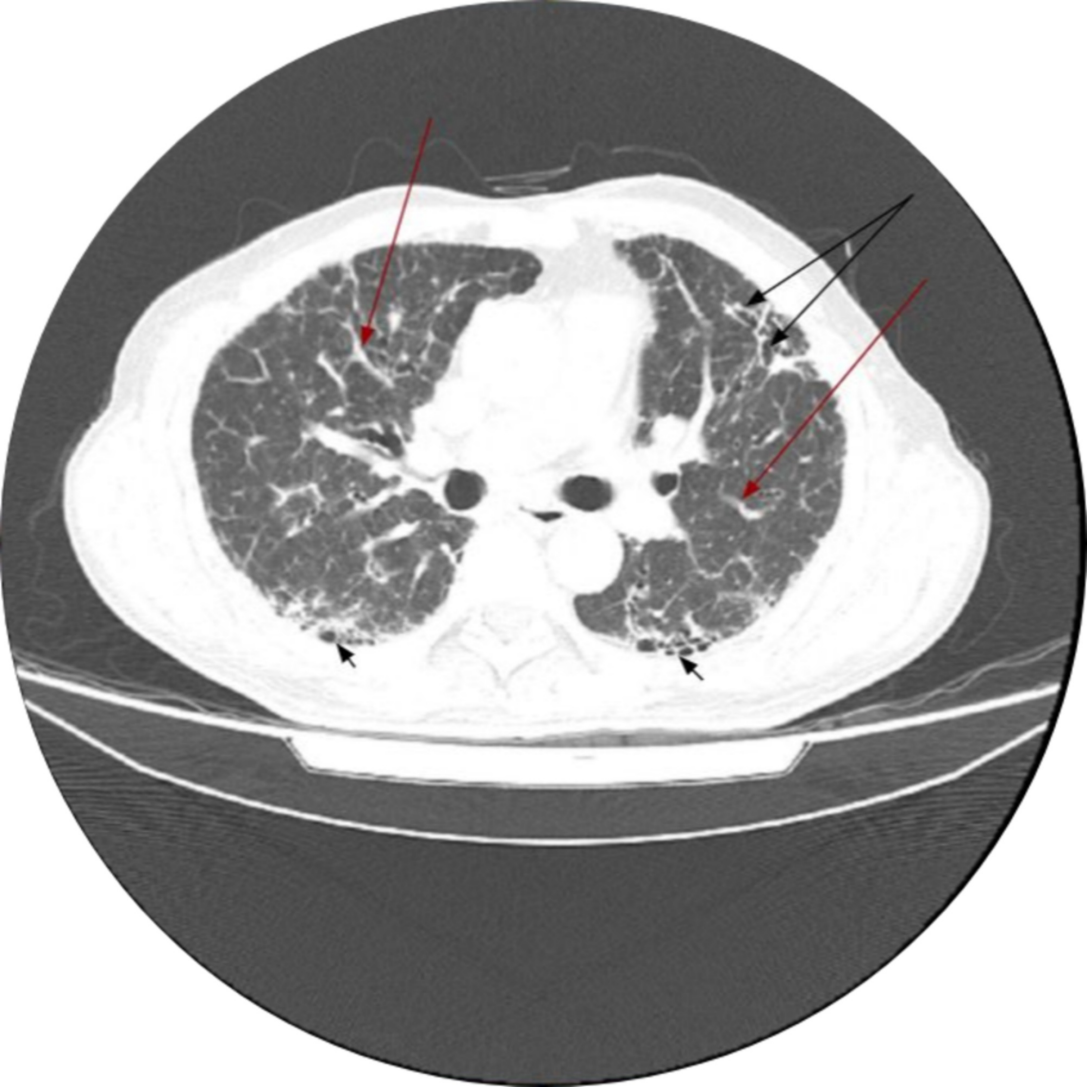
High-resolution computed tomographic features of usual interstitial pneumonia Diffuse interlobar interstitial thickening is noted at bilateral aerated lungs (*red arrows*) along with honeycombing more prevalent at lung bases (*short black arrows*) and mild traction bronchiectasis at left upper lobe (*longer black arrows at the top*)

Over the last nine months, he had exertional dyspnea and a dry cough. He experienced no constitutional symptoms like low-grade fever, fatigue, malaise or weight loss. He had no past medical history of recurrent episodes of sinusitis or respiratory tract infections, or gastroesophageal reflux disease. He was on home oxygen to maintain a peripheral oxygen saturation (SpO_2_) above 90% and pirfenidone therapy, and had adhered to his medical regime. Apart from that, he was taking no other medications. He had smoked one pack of cigarettes daily for 15 years and quit 20 years ago. The patient worked as an investment banker and did not use alcohol or illicit drugs. There was no exposure to an inciting agent (eg birds, pets, fungi, chemicals). He had not traveled recently, but was caring for his wife since four days who had tested positive for COVID-19. His last high-resolution computed tomography (HRCT) done a week before presentation has been included for analysis; a definite usual interstitial pneumonia pattern on this HRCT affirmed IPF (Figure 1) [6]. 

**Figure 2:**
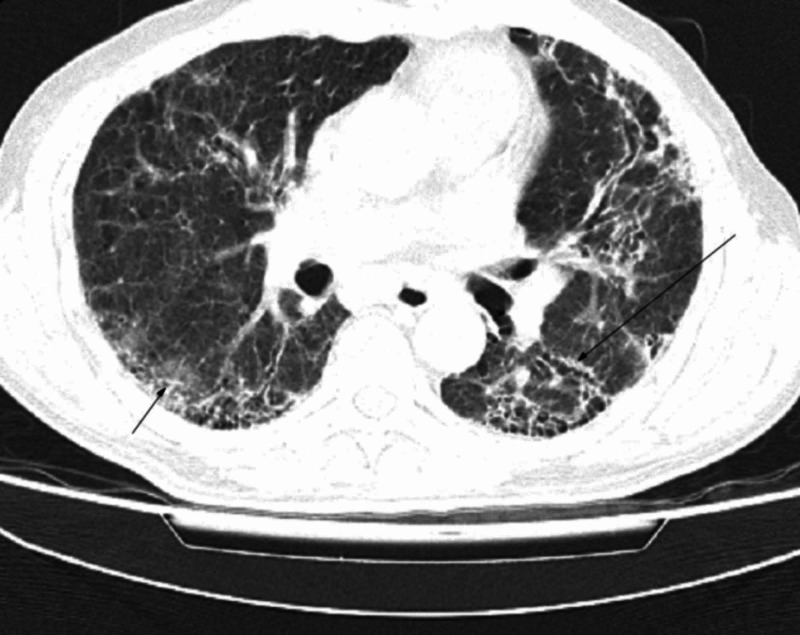
High-resolution computed tomography scan of a COVID-19 patient with preexisting idiopathic pulmonary fibrosis New-onset bilateral ground-glass haze (*black arrows*) superimposed on underlying usual interstitial pneumonia

When checked on admission to the Isolation Chamber, his temperature was 40 C, blood pressure was 70/40 mmHg, pulse was 130/min and regular, and respirations were 40/min. The patient was lethargic and confused. Physical examination revealed conjunctival pallor and an unremarkable jugular venous pulse, and no evidence of joint disease. Bilateral crackles were heard on lung auscultation. Cardiac auscultation revealed no murmurs or additional sounds. Pulse oximetry showed 90% SpO_2_ on room air. His real-time RT-PCR assay of nasal and pharyngeal swab specimens for SARS-CoV-2 had come out positive. HRCT done at this time revealed new ground-glass opacities superimposed on pulmonary fibrosis (Figure 2). No cavitating lesion, soft tissue attenuation nodule(s), pleural effusion or hilar adenopathy was observed and there was no evidence of discrete chamber or main vessel enlargement. Treatment was initiated with cap hydroxychloroquine 400 mg × BD, IV azithromycin 400 mg × OD, IV solu cortef 100 mg × TDS, and heparin. He was also being treated with IV fluids and a norepinephrine infusion.

Workup for autoimmune/rheumatological diseases (antinuclear antibodies, rheumatoid factor, anti dsDNA, anti-cardiolipin antibodies, antiphospholipid antibodies etc) was already negative. His C-reactive protein was 55.9 mg/L, ferritin was 1480 ng/mL (normal: 15-200 ng/mL), and lactate dehydrogenase was 443 U/L (normal: 122-222 U/L). Coagulation studies revealed a prothrombin time (PT) of 23 sec, activated partial thromboplastin time (aPTT) of 53 sec, and D-dimer level of 2.1 μg/dL (normal: <0.5 μg/dL). Blood count, serum chemistry and liver function study is given in Table 1.

**Table 1:** Blood count, liver function tests, renal function tests and serum electrolytes These reference range values are typical for adult men aged 60 years or above. Normal results vary from laboratory to laboratory and might be slightly different for women and children. Abbreviations: HCT, hematocrit; MCV, mean corpuscular volume; ALT, alanine aminotransferase; AST, aspartate aminotransferase; BUN, blood urea nitrogen

	Reference range	Patient’s value	Comments
Leukocyte count	3.4-9.6×​​​​​​10^3^/μL	3.3×​​​​​​10^3^/μL	Low
Lymphocytes #	1.00-4.80×​​​​​​10^3^/μL	0.8×​​​​​​10^3^/μL	Low
Erythrocyte count	4.32-5.72×​​​​​​10^6^/μL	2.99×​​​​​​10^6^/μL	Low
Hemoglobin	13.2-16.6 g/dL	8.2 g/dL	Low
HCT	38.3-48.6%	24.4%	Low
MCV	80-100 fL	81.7 fL	Normal. Low Hemoglobin with normal MCV strongly suggestive of hemolytic anemia
Platelet Count	135-317×​​​​​​10^3^/μL	71×​​​​​​10^3^/μL	Low
ALT, serum	7-55 U/L	156 U/L	High
AST, serum	8-48 U/L	65 U/L	High
BUN, serum	7-20 mg/dL	60 mg/dL	High
Creatinine, serum	0.84-1.21 mg/dL	1.8 mg/dL	High
Sodium, serum	135-145 mEq/L	134 mEq/L	Slightly low
Potassium, serum	3.6-5.2 mEq/L	5.2 mEq/L	Higher side of normal
Chloride, serum	95-105 mEq/L	104 mEq/L	Normal

Arterial blood gas status six hours into admission is given in Table 2. Decreased bicarbonate level and increased anion gap (20.1 mEq/L) were indicative of anion-gap metabolic acidosis in the patient.

**Table 2:** Arterial blood gas

	Patient’s value	Comments
pH	7.035	Low
paCO_2_	37.0 mm Hg	Normal
HCO_3_-	9.9 mEq/L	Low

On the night following admission, his hemodynamic status deteriorated rapidly. Blood pressure fell to 50/20 mmHg despite receiving IV fluids. The venous access sites were oozing blood. Urine drainage from the Foley catheter was <20 mL/hr. GCS fell to 4/15. SpO_2 _on pulse oximeter was 86%. The patient was put on high flow oxygen, and remained on double inotropic support and Inj dopamine at 3 µg/kg/min. But despite all efforts, he collapsed of overwhelming infection. The emergency medical team found the patient unresponsive with no pulse.

## Discussion

This patient's high fever, lymphocytopenia are indicative of SARS-CoV-2 pneumonia complicated by septic shock (fluid-refractory hypotension, tachycardia), the result of an overwhelming inflammatory response to infection. Severe sepsis is marked by organ dysfunction due to poor blood flow (eg oliguria from poor renal perfusion and altered mental status from poor cerebral perfusion). His laboratory studies showed a consumptive coagulopathy (thrombocytopenia, prolonged PT/aPTT), which, along with bleeding from the IV catheter site and elevated D-dimer, was likely due to DIC [7].

IPF patients have an inexorable decline in respiratory reserve and lung function. Cytokines are implicated in the pathogenesis of IPF including initiation of a process called epithelial-mesenchymal transition, and SARS-CoV-2 itself causes further cytokine activation and inflammation thereby also justifying the use of steroids as a therapeutic option in virally driven hyperinflammation, but precisely why COVID-19-linked sepsis may be fatal [2,8]. HRCT is a sensitive diagnostic tool for SARS-CoV-2 which may manifest itself as a worsening of the fibrotic and inflammatory process in IPF with the appearance of new bilateral ground-glass opacities that have a propensity toward the basilar lung segments. All this can further be studied upon.

Answers to the following questions are being sought: the impact of COVID-19 on the rate of preexisting ILD progression, the mortality rate and predictors of mortality in patients with preexisting ILD and COVID-19 [9].

In an analysis issued by the Chinese Center for Disease Control and Prevention using a cross-sectional study design, among the 44,672 confirmed cases of COVID-19, the case fatality rate was found to be elevated in those aged >/60 years, in males and among those with preexisting comorbids-10.5% for cardiovascular disease, 7.3% for diabetes, 6.3% for chronic respiratory disease, 6% for hypertension, and 5.6% for cancer. All 1023 deaths that occurred were critical cases; critical cases were identified as those that exhibited respiratory failure, septic shock, and/or multiple organ dysfunction [10]. In a comprehensive review that also looked at the comorbid in five different COVID-19 studies from China for comparison, for a total of 409 patients with comorbidity, coexisting chronic obstructive pulmonary disease was identified in 27 [11]. There is far less detail in literature specifically about IPF.

In a retrospective study to determine the clinical outcome in 38 patients with a mean age of 68.3+/-11.5 years with IPF admitted to the Intensive Care Unit between 1995 and 2000, of the 37 patients whose data was available, nine patients (24%) developed sepsis with one patient each developing severe sepsis and septic shock. Thirty-five patients (95%) developed one or more organ failure. IPF was associated with poor short and long term prognosis [12]. Although sepsis has been cited as a complication in those with IPF, to my knowledge there is only a single case report so far detailing COVID-19 sepsis in a patient with a history of IPF [13]. The outcome of the patient was fatal in that case too.

## Conclusions

SARS-CoV-2 has a propensity to affect older adults with underlying IPF. IPF patients are at high risk for invasive and potentially fatal SARS-CoV-2 pneumonia. Manifestations of sepsis are more acute and severe in IPF patients. IPF patients be alerted to their increased susceptibility to fulminant SARS-CoV-2 infection and therefore they must go an extra mile to reduce potential exposure to COVID-19. Research is needed to elucidate further the clinical course, diagnostic criteria and outcomes of patients with concurrent COVID-19 and IPF.
